# Have we been guilty of ageism in the primary treatment of breast cancer?

**DOI:** 10.1038/sj.bjc.6603697

**Published:** 2007-04-03

**Authors:** K I Pritchard

**Affiliations:** 1 Toronto-Sunnybrook Regional Cancer Centre, Clinical Trials and Epidemiology, 2075 Bayview Avenue, Toronto, Ontario, Canada M4N 3M5

The current issue of the British Journal of Cancer contains a well-conducted meta-analysis of six small randomised trials comparing appropriate surgery, with or without tamoxifen to tamoxifen alone as primary treatment for elderly women with operable breast cancer. The meta-analysis included 517 women treated in studies of surgery plus tamoxifen compared to tamoxifen alone, and 247 women treated in studies of surgery compared to tamoxifen alone. A meta-analysis of each subgroup showed significant improvement in progression-free survival (hazard ratio (HR)=0.55; 95% confidence interval (CI) 0.39–0.77; *P*=0.006) for surgery in comparison to adjuvant tamoxifen and 0.65 (95% CI 0.53–0.81; *P*=0.0001) for surgery plus tamoxifen in comparison to tamoxifen alone. Overall survival was not improved in the meta-analysis of surgery alone compared to tamoxifen alone, but was marginally significantly better for adjuvant tamoxifen plus surgery compared to adjuvant tamoxifen alone (HR=0.86; 95% CI, 0.83–1; *P*=0.06). Interestingly, a previous meta-analysis by Mustacchi *et al* (1994) also suggested a marginally improved risk ratio of 0.86 (*P*=0.09) for adjuvant tamoxifen and surgery in comparison to tamoxifen alone for overall survival and 0.70 (*P*<0.05) for breast cancer-related survival.

The paradigm that women aged 70 and over might receive primary treatment for breast cancer with tamoxifen or other endocrine therapy alone, based on the concept that they are less fit for surgery because of age and co-morbidity, was developed in the 1980s and has been since then considered appropriate to a greater or lesser degree in various countries. Particularly in the United Kingdom, this approach, apparently, has been used in as many as 42% of women in this age group regardless of whether co-morbidity was present or not (Wyld *et al*, 2004).

This meta-analysis however, and that previously published by Mustacchi, as well as a re-examination of the life expectancy of women in their 70s today, would suggest that to approach these women differently from their younger sisters may be quite inappropriate.

In fact, the annual incidence of breast cancer increases with age, and divided by decade, more women are diagnosed with breast cancer in the combined decades of their 70s and 80s than in their 50s or their 60s. Unfortunately, most studies of breast cancer therapy worldwide have, at least until recently, specifically excluded women over 70 or even over 65, in itself a form of ageism.

In addition, physicians often tend to underestimate life expectancy in elderly women. Although the life expectancy of women born today in most countries of the developed world is well over 70, women who have already reached their 70s are of course likely to live to be even older. Today in Canada, the average life expectancy for a woman of 70 without particular morbidity is more than 16 years and that for a 75-year-old is almost 13 years (Ottawa, 2006). The median time to progression in women in the studies included in this meta-analysis who received only endocrine therapy is not available in this publication; however, Kenny *et al* (1998) reported that whereas 70% of those receiving surgery alone were free of local disease at 24 months, only 47% of those receiving tamoxifen alone were free of local disease (Kenny *et al*, 1998). Similarly, the GRETA trial showed that local progression at 36 months was 25% for primary tamoxifen and only 6% for surgery plus tamoxifen. These numbers suggest that local control with tamoxifen alone is quite inadequate for women with this life expectancy.

The only available quality of life data suggests that in one study, 3 months after the start of treatment, the surgery group had more psychosocial morbidity (*P*=0.03), but that there was no difference between the two groups at 2 years (Fallowfield *et al*, 1994).

Another factor that contributes to undertreatment in women in this age group is that older women may be less likely to seek information, or to be assertive about therapy choices. They may also, on occasion, feel that they cannot undertake hospital admission or protracted treatment such as daily radiation therapy because of their responsibilities in caring for an elderly partner. Today however, with the availability of effective conservative surgery including breast conserving surgery, and sentinel node dissection with nodal sampling, even elderly patients probably require minimal hospitalisation if offered appropriate homecare support following their surgery. As stated by Hind *et al*, both mastectomy and wide local excision have, for several decades, had low mortality rates (Hunt *et al*, 1980; Wyld and Reed, 2004). Furthermore, modern anaesthesia methods should reduce surgical risk even in those with accompanying co-morbidity. Although breast surgery-related morbidity may impact on quality of life, if only lumpectomy or lumpectomy and sentinel node dissection is used, such morbidity could be minimised.

Complete axillary dissection should only be necessary in those with involved nodes at sentinel node sampling. Axillary dissection is generally considered to contribute to local control, to guide the selection of systemic therapy, and perhaps to have some long-term effect on survival, although with competing risks of death in this patient population such an effect, if any, would be minor. If the tumour is large enough and of high enough histologic risk to require systemic therapy in any case, axillary dissection or sampling might not be required except for reasons of local control. This may be decided on an individual basis. In the case of lumpectomy, radiation therapy is actually generally well tolerated in the elderly. Daily visits may be required, but with a variety of brachytherapy approaches being explored, even this may not be necessary. Ongoing trials of chemotherapy in this age group (Biganzoli *et al*, 2004; Muss *et al*, 2005) may also help us to see whether adjuvant chemotherapy will provide more gains than negative effects for these older women.

In conclusion, to approach women in their 70s without seriously considering the potential positive benefit of appropriate surgery including complete tumour resection and axillary sampling, and consideration of radiation therapy, as well as adequate systemic therapy, would seem inappropriate. Has this policy been perhaps a method of rationing care? – or a thoughtless application of ageism? In fact, what would be appropriate for the 50-year-old may be equally appropriate for the 70-year-old.

So, it would seem that treatment decisions in women over 70 should be made more in relation to their general health than to their age, and should be similar to those made in women in their 50s and 60s unless serious co-morbidities exist. More trials involving this group of women should be designed and carried out in order to have better data to use for decision-making in these women. Although apparently up to 40% of women over 70 have been treated with endocrine therapy alone in the UK, fewer than 1000 became part of the randomised trials examining this approach. However, these trials, although small, have been pivotal, in that their combination in meta-analysis has allowed us to critically examine and hopefully improve our approach to these patients.

With otherwise healthy women of 70 expected to live well into their 80s and perhaps 90s, we must abandon ageist approaches in the treatment of breast cancer as well as in other areas of medical care. Remember…70 is the new 50!
